# Elevated serum nuclear factor erythroid 2-related factor 2 levels contribute to a poor prognosis after acute supratentorial intracerebral hemorrhage: A prospective cohort study

**DOI:** 10.3389/fnagi.2022.1014472

**Published:** 2022-10-24

**Authors:** Chuan-Liu Wang, Xin-Jiang Yan, Cheng-Liang Zhang, Yan-Wen Xu

**Affiliations:** ^1^Department of Neurology, The Quzhou Affiliated Hospital of Wenzhou Medical University, Quzhou People’s Hospital, Quzhou, Zhejiang, China; ^2^Department of Neurosurgery, The Quzhou Affiliated Hospital of Wenzhou Medical University, Quzhou People’s Hospital, Quzhou, Zhejiang, China

**Keywords:** nuclear factor-erythroid 2-related factor 2, intracerebral hemorrhage, early neurologic deterioration, outcome, severity, mechanism

## Abstract

**Objective:**

Nuclear factor erythroid 2-related factor 2 (Nrf2) is a key transcriptional factor for antioxidant response element-regulated genes. The purpose of this study was to assess the prognostic role of serum Nrf2 in intracerebral hemorrhage (ICH).

**Materials and methods:**

In this prospective observational study, serum Nrf2 levels of 115 acute supratentorial ICH patients and 115 controls were gaged. Early neurologic deterioration (END) was defined as an increase of four or greater points in National Institutes of Health Stroke Scale (NIHSS) score or death at post-stroke 24 h. A poor outcome was referred to as the post-stroke 90-day modified Rankin scale (mRS) score of 3–6. END and a poor outcome were considered as the two prognostic parameters.

**Results:**

As compared to controls, serum Nrf2 levels of patients were substantially elevated (*P* < 0.001), with its levels increasing during the 6-h period immediately, peaking in 12–18 h, plateauing at 18–24 h, and decreasing gradually thereafter (*P* < 0.05). Serum Nrf2 levels of patients were independently correlated with NIHSS score (*t* = 3.033; *P* = 0.003) and hematoma volume (*t* = 3.210; *P* = 0.002), independently predicted END (odds ratio 1.125; 95% confidence interval 1.027–1.232; *P* = 0.011) and poor outcome (odds ratio 1.217; 95% confidence interval 1.067–1.387; *P* = 0.013), as well as efficiently distinguished END (area under curve 0.771; 95% confidence interval 0.666–0.877; *P* < 0.001) and poor outcome (area under curve 0.803; 95% confidence interval 0.725–0.882; *P* < 0.001). Its predictive ability was equivalent to those of NIHSS score and hematoma volume (both *P* > 0.05), and it also significantly improved their predictive abilities under receiver operating characteristic (ROC) curve (all *P* < 0.05).

**Conclusion:**

Elevated serum Nrf2 levels are closely correlated with severity, END, and 90-day poor outcome following ICH. Hence, Nrf2 may play an important role in acute brain injury after ICH, and serum Nrf2 may have the potential to serve as a prognostic biomarker of ICH.

## Introduction

Spontaneous intracerebral hemorrhage (ICH) is an acute life-threatening cerebrovascular disease that features neurologic deficits and even a decline in consciousness (Hemphill et al., 2015). National Institutes of Health Stroke Scale (NIHSS) is a conventional indicator of neurologic function (Kwah and Diong, 2014), and hematoma volume is a common variable for assessing ICH radiological severity (LoPresti et al., 2014). Early neurologic deterioration (END) is a frequently encountered adverse event, which has potentially serious consequences on patient outcome (Leira et al., 2004). Hence, early distinguishing ICH patients at risk of END and a poor outcome can have considerable clinical significance.

Oxidative stress arising from intraparenchymal blood deposited during ICH is extensively accepted as the important pathophysiology of secondary brain injury after ICH (Masomi-Bornwasser et al., 2021). During recent decades, biomarkers have obtained researchers’ interests with respect to ICH severity assessment and prognosis prediction (Cai et al., 2021; Wu et al., 2021). Clearly, some oxidative stress-related biomarkers such as malondialdehyde, myeloperoxidase, lipid hydroperoxide, 8-iso-prostaglandin F2α, and thioredoxin have been demonstrated to be highly associated with ICH severity and clinical outcomes (Alexandrova and Danovska, 2011; Du et al., 2014; Qian et al., 2016; Lorente et al., 2018; Zheng et al., 2018). Nevertheless, those biomarkers in the peripheral blood have not been routinely determined for clinical service. Consequently, a clinical investigation of biochemical markers is still underway for ICH severity and prognosis analysis.

Nuclear factor erythroid 2-related factor 2 (Nrf2) has been extensively investigated as a very important cytoprotective transcription factor that can regulate the expression of genes coding antioxidant, anti-inflammatory, and detoxifying proteins (Pi et al., 2019). Nrf2 by neurons can be highly expressed under brain oxidative stress and inflammation injury (Sandberg et al., 2014). Compelling experimental evidence has confirmed that Nrf2 may confer brain-protective function *via* attenuating free radical oxidative damage in ICH (Wang et al., 2007; Zhao et al., 2007, 2015a,2015b). Taken together, circulating Nrf2 is hypothesized to represent a potential biomarker of ICH. Recently, in autism children, serum Nrf2 levels were found to be significantly elevated, as compared to healthy children (Ayaydin et al., 2020). However, to the best of our knowledge, there is a paucity of data available regarding Nrf2 levels in the peripheral blood of humans with acute brain injury, including ICH. In this study, we determined serum Nrf2 levels in a cohort of patients with spontaneous supratentorial ICH and compared them with those of controls. In addition, we investigated the role of serum Nrf2 as a potential biomarker for severity assessment and prognosis prediction of ICH.

## Materials and methods

### Study design ad participant selection

Patients with first-ever spontaneous supratentorial ICH were consecutively recruited into this prospective observational study performed at our hospital from February 2018 to July 2021. Next, we excluded those patients with (1) time from the onset of stroke symptom to hospital admission >24 h; (2) age <18 years; (3) ICH resulting from secondary causes (such as congenital or acquired coagulation abnormalities, hemorrhagic transformation of cerebral infarction, moyamoya disease, cerebral aneurysm, cerebral arteriovenous malformation, and intracranial tumors); (4) primary intraventricular hemorrhage; (5) a surgical procedure for hematoma evacuation; or (6) a history of some specific diseases or conditions (e.g., ischemic stroke, aneurysmal subarachnoid hemorrhage, intracranial tumors, severe traumatic brain injury, malignancies, immune deficiency syndromes, pregnancies, and severe heart, liver, lung, or kidney dysfunction). During the same period, a group of healthy subjects were recruited as controls. This study was performed according to the tenets of the Declaration of Helsinki, and the approval for the protocol of this study was acquired from the ethics committee at our hospital. Informed consent to participate in this study was signed by next of kin to patients or controls themselves.

### Data collection and outcome assessment

We collected some relevant information such as demographics (age and gender), adverse life habits (cigarette smoking and alcohol drinking), previous usage of some specific drugs (statins, anticoagulation drugs, and antiplatelet drugs), vascular risk factors (hypertension, diabetes mellitus, and hyperlipidemia), and vital signs (systolic and diastolic arterial blood pressures). Admission NIHSS scores were recorded to assess neurologic function. Hematoma volume was calculated based on the formula 0.5 × a × b × c (Kothari et al., 1996). The ICH topography was classified as lobar, including parietal, frontal, temporal, and occipital, when it affected predominantly the cortical or subcortical white matter of the cerebral lobes or as deep, including putamen, caudate, internal capsule, and thalamus, when it was limited to the internal capsule, the basal ganglia, or the thalamus. Extension of hematoma into subarachnoid or intraventricular cavity was observed. The severity of intraventricular hematoma was estimated utilizing the Graeb scale (Graeb et al., 1982). END was defined as an increase of ≥4 in the NIHSS score or death within 24 h after admission (Du et al., 2013). A poor outcome was defined as post-stroke 90-day modified Rankin scale (mRS) scores of 3–6 (Dong et al., 2015).

### Immune analysis

Five milliliters of venous blood was drawn *via* the antecubital vein from ICH patients and healthy controls and promptly placed into gel-containing biochemistry tubes. The blood samples were immediately centrifuged at 3,500 *g* for 10 min. Serum was preserved at −80°C in Eppendorf tubes until assayed. Serum Nrf2 levels were quantified in the biochemistry laboratory using the enzyme-linked immunosorbent assay (Active Motif, Carlsbad, CA, USA) and read at 450 nm on a microplate reader (Multiskan GO, Thermo Fisher Scientific, Waltham, MA, USA). Each sample was in duplicate measured by the same technician blinded to clinical data, the results were reported as ng/ml, and two measures were averaged for final analysis.

### Statistical analysis

Statistical analysis was performed using the Statistical Package for Social Sciences (SPSS) 23.0 software (SPSS Inc., Chicago, IL, USA). The Pearson Chi-square test was applied to compare the proportions of categorical variables, which were herein reported as frequencies (percentages). The Shapiro–Wilk normality test was used to assess normal distribution of continuous variables, which were therein summarized as medians (upper–lower quartiles) if non-normally distributed and as means (standard deviation, SD) if normally distributed. If the continuous data were not normally distributed, we used non-parametric tests, including the Mann–Whitney U test for two-group comparisons and the Kruskal–Wallis test for multiple-group comparisons. If the continuous data were under normal distribution, the independent sample *t*-tests were utilized for two-group comparisons. Spearman’s correlation coefficient was applied to evaluate the relationships between serum Nrf2 levels and other variables, and afterward, linear regression analysis was performed. The binary logistic regression model was established to discern predictors, which were independently associated with END and 90-day poor outcome after ICH. Associations were reported as odds ratio (OR) with the corresponding 95% confidence interval (CI). In this study, those variables, which were significant using univariate analysis, were forced into the multivariate model. Under the receiver operating characteristic (ROC) curve, we observed predictive efficiency. The area under ROC curve (AUC) was shown to reflect prognostic ability. The two-sided significance level was set at *P* < 0.05.

## Results

### Participant selection

**FIGURE 1:**
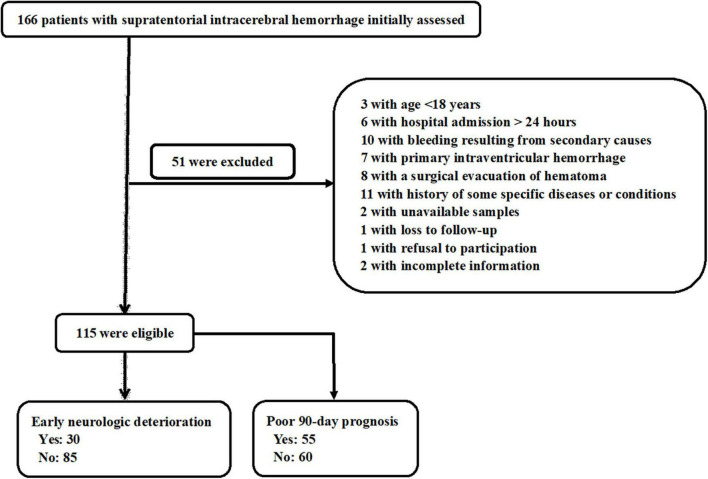
Flowchart for selecting eligible patients with acute supratentorial intracerebral hemorrhage (ICH). After 51 were excluded from 166 patients, a total of 115 patients were retained for final analysis.

In this study, a total of 166 patients with first-ever supratentorial ICH were assessed for eligibility, of whom 51 patients were excluded from this study because of the reasons outlined in Figure 1 and 115 patients were finally analyzed. Also, 115 healthy subjects constituted controls. Patients were aged from 37 to 86 years (mean 62.9 years; SD 12.0 years), of whom 65 were men and 50 were women. Controls, 60 being men and 55 being women, were aged from 30 to 93 years (mean 63.3 years; SD 13.7 years). There were no substantial differences in age and gender percentage between controls and patients (both *P* > 0.05). Other baseline characteristics are provided in Table 1 for comparison between ICH patients and healthy controls. Also, alcohol drinking and cigarette smoking did not significantly differ between ICH patients and healthy controls (both *P* > 0.05).

**TABLE 1:** Comparison of the baseline characteristics between healthy controls and patients with intracerebral hemorrhage (ICH).

	Patients	Controls	*P*-value
Age (years)	62.9 ± 12.0	63.3 ± 13.7	0.770
Gender (male/female)	65/50	60/55	0.508
Hypertension	73 (63.5%)	–	
Diabetes mellitus	18 (15.7%)	–	
Hyperlipidemia	33 (28.7%)	–	
Cigarette smoking	39 (33.9%)	35 (30.4%)	0.572
Alcohol drinking	44 (38.3%)	42 (36.5%)	0.785

Variables were presented as count (percentage) or mean ± standard deviation as appropriate, and statistical methods included the Chi-square test, Fisher’s exact test, and Student’s *t*-test.

### Patient characteristics

Among this cohort of patients, 73 (63.5%) presented with hypertension, 18 (15.7%) had diabetes mellitus, 33 (28.7%) suffered from hyperlipidemia, 39 (33.9%) were cigarette smokers, and 44 (38.3%) were alcohol consumers. In total, prior to hospital admission, 25 (21.7%), 6 (5.2%), and 16 (13.9%) patients orally took statins, anticoagulation drugs, and antiplatelet drugs, respectively. Patients were admitted from 0.5 to 24.0 h (median 10.0 h; lower–upper quartiles 6.9–14.8 h) following ICH. Systolic arterial pressure and diastolic arterial pressure ranged from 98 to 214 mmHg (mean 150.2 mmHg; SD 23.8 mmHg) and from 65 to 114 mmHg (mean 87.1 mmHg; SD 10.9 mmHg), respectively. Lobar hematomas and deep hematomas were revealed in 26 and 89 patients, respectively. Hematomas were extended into intraventricular cavity in 38 patients (33.0%) and into subarachnoid space in 12 patients (10.4%). NIHSS scores, Graeb scores, and bleeding size ranged from 0 to 18 ml (median 7 ml; lower–upper quartiles 4–11 ml), from 0 to 9 ml (median 0 ml; lower–upper quartiles 0–1 ml), and from 3 to 46 ml (median 13 ml; lower–upper quartiles 7–22 ml), respectively.

### Serum nuclear factor erythroid 2-related factor 2 levels after intracerebral hemorrhage

**FIGURE 2:**
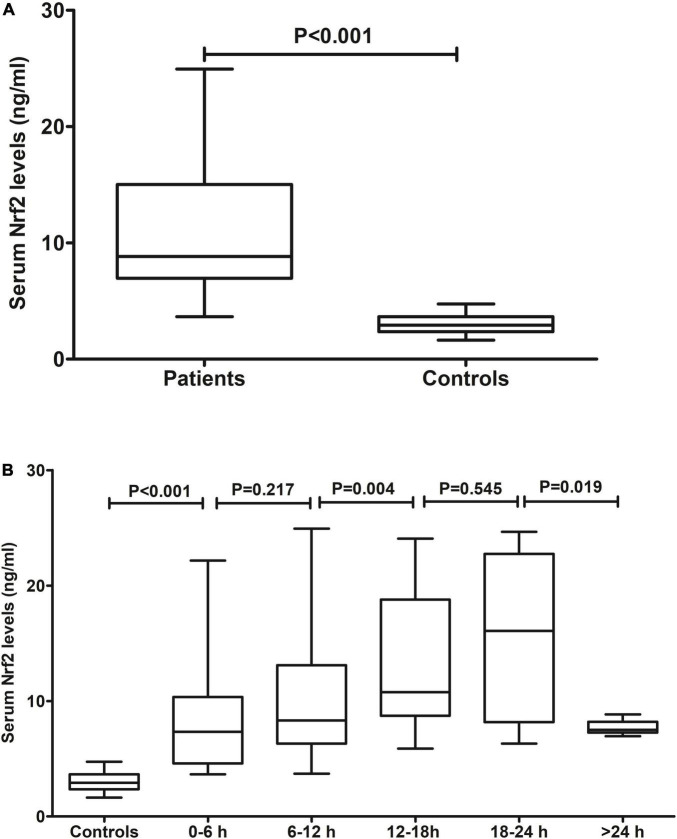
Serum nuclear factor erythroid 2-related factor 2 (Nrf2) levels after acute intracerebral hemorrhage (ICH). **(A)** Difference in serum Nrf2 levels between controls and patients. **(B)** Dynamic change in serum Nrf2 levels after acute ICH. After stroke, serum Nrf2 levels of patients increased during the 6-h period immediately, peaked at 12–18 h, plateaued at 18–24 h, decreased gradually thereafter, and were significantly higher than those in healthy controls (all *P* < 0.05). Nrf2 indicates nuclear factor erythroid 2-related factor 2.

Blood samples of patients were collected from 1.0 to 26.0 h (median 11.5 h; lower–upper quartiles 7.8–16.4 h) after ICH. In Figure 2A, serum Nrf2 levels were substantially higher in patients than those in controls (*P* < 0.001). In addition, blood collection time was divided into five time points, namely, 0–6 h, 6–12 h, 12–18 h, 18–24 h, and >24 h. After ICH, serum Nrf2 levels of patients increased during the 6-h period immediately, peaked at 12–18 h, plateaued at 18–24 h, decreased gradually thereafter, and were significantly higher than those in healthy controls (all *P* < 0.05; Figure 2B).

### Serum nuclear factor erythroid 2-related factor 2 levels and illness severity after intracerebral hemorrhage

**FIGURE 3:**
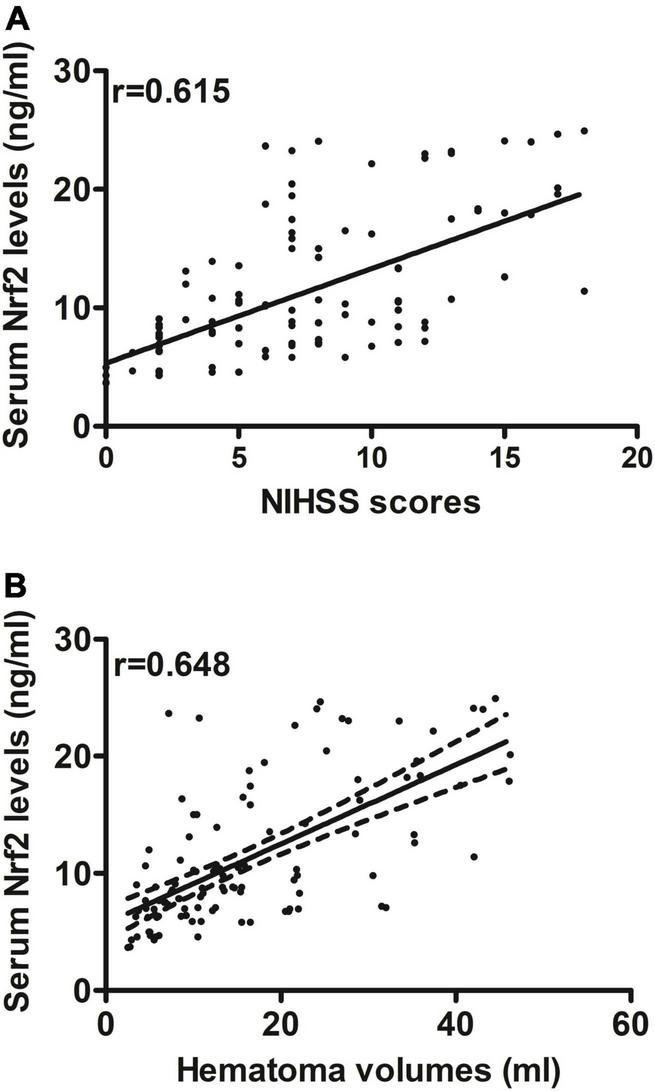
Relationship between serum nuclear factor erythroid 2-related factor 2 (Nrf2) levels and stroke severity after acute supratentorial ICH. **(A)** Correlation of serum Nrf2 levels with National Institutes of Health Stroke Scale (NIHSS) score. **(B)** Correlation of serum Nrf2 levels with hematoma volume. Serum Nrf2 levels were closely and positively correlated with NIHSS score and hematoma volume (both *P* < 0.001). Nrf2 indicates nuclear factor erythroid 2-related factor 2; NIHSS, National Institutes of Health Stroke Scale.

Serum Nrf2 levels of patients were intimately correlated with NIHSS scores (*P* < 0.001; Figure 3A), hematoma volume (*P* < 0.001; Figure 3B), and other variables, including admission time, blood collection time, intraventricular hemorrhage, Graeb scores, subarachnoid hemorrhage, blood glucose levels, and blood leukocyte count (all *P* < 0.05; Table 2). Using the multivariate linear regression model, which contained significant variables in the Spearman test, it was shown that serum Nrf2 levels were independently related to NIHSS scores (*t* = 3.033; *P* = 0.003) and hematoma volume (*t* = 3.210; *P* = 0.002).

**TABLE 2:** Factors correlated with serum nuclear factor erythroid 2-related factor 2 (Nrf2) levels after acute intracerebral hemorrhage (ICH).

Variables	*r*	*P*-value
Age >60 years	0.092	0.327
Gender (male/female)	–0.059	0.532
Hypertension	0.082	0.381
Diabetes mellitus	0.162	0.084
Hyperlipidemia	0.058	0.539
Cigarette smoking	0.165	0.077
Alcohol drinking	0.099	0.293
Pretreatment of statins	0.097	0.305
Pretreatment of anticoagulation drugs	–0.168	0.073
Pretreatment of antiplatelet drugs	0.114	0.224
Admission time (h)	0.211	0.024
Blood collection time (h)	0.220	0.018
Systolic arterial pressure (mmHg)	0.068	0.469
Diastolic arterial pressure (mmHg)	0.092	0.330
Hemorrhage locations (lobar/deep)	0.035	0.712
Intraventricular hemorrhage	0.477	< 0.001
Graeb scores	0.430	< 0.001
Subarachnoid hemorrhage	0.279	0.003
NIHSS scores	0.615	< 0.001
Hematoma volume (ml)	0.648	< 0.001
Blood leukocyte count (×10^9^/l)	0.439	< 0.001
Blood glucose levels (mmol/l)	0.279	0.003

Correlations were analyzed using Spearman’s correlation coefficient test, and the results were reported as *r* values. NIHSS indicates National Institutes of Health Stroke Scale.

### Serum nuclear factor erythroid 2-related factor 2 levels and early neurologic deterioration risk

**FIGURE 4:**
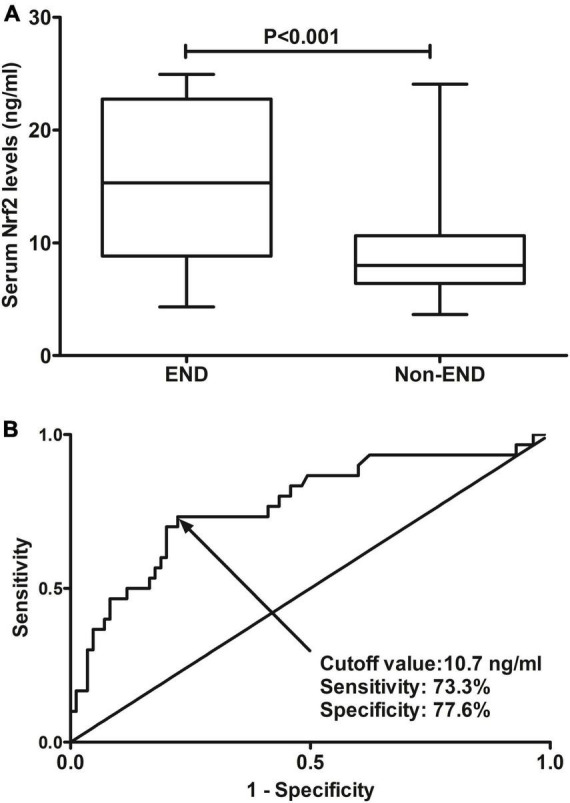
Predictive ability with respect to serum nuclear factor erythroid 2-related factor 2 (Nrf2) levels for risk of early neurologic deterioration (END) after acute supratentorial intracerebral hemorrhage (ICH). **(A)** Comparison of serum Nrf2 levels between patients with END and those who did not present with END. **(B)** Discriminatory capability of serum Nrf2 levels for END under the receiver operating characteristic (ROC) curve. Serum Nrf2 levels were markedly higher in patients suffering from END than in those without development of END (*P* < 0.001). Serum Nrf2 levels statistically significantly predicted END after acute stroke (*P* < 0.001). Nrf2 indicates nuclear factor erythroid 2-related factor 2; END, early neurologic deterioration.

**FIGURE 5:**
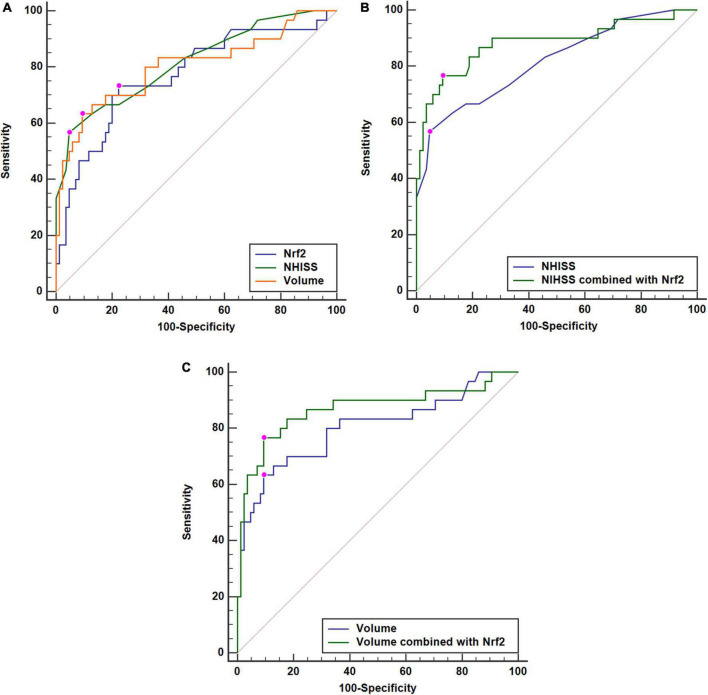
Comparisons of discriminatory capabilities for early neurologic deterioration (END) among serum nuclear factor erythroid 2-related factor 2 (Nrf2) levels, National Institutes of Health Stroke Scale (NIHSS) scores, and hematoma volume after acute supratentorial intracerebral hemorrhage (ICH). **(A)** Comparisons of areas under curve for END among serum Nrf2 levels, NIHSS scores, and hematoma volume after acute supratentorial ICH. **(B)** Comparison of areas under curve for END between serum Nrf2 levels combined with NIHSS scores and NIHSS scores after acute supratentorial ICH. **(C)** Comparison of areas under curve for END between serum Nrf2 levels combined with hematoma volume and hematoma volume after acute supratentorial ICH. Serum Nrf2 levels had similar area under curve, as compared to NIHSS scores and hematoma volume (both P > 0.05). Moreover, serum Nrf2 levels profoundly improved areas under curve of NIHSS scores and hematoma volume (both *P* < 0.05). Nrf2 indicates nuclear factor erythroid 2-related factor 2; NIHSS, National Institutes of Health Stroke Scale.

A total of 30 ICH patients experienced END. In Figure 4A, patients at risk of END had significantly higher serum Nrf2 levels than those without development of END (*P* < 0.001). Using Youden’s method, serum Nrf2 levels >10.7 ng/ml discriminated patients at risk of END with medium–high sensitivity and specificity values (Figure 4B). In Figure 5A, using the ROC curve, the predictive ability of serum Nrf2 levels was in the range of NIHSS scores and hematoma volume (both *P* > 0.05). Moreover, serum Nrf2 levels significantly improved the predictive values of NIHSS scores (*P* = 0.030; Figure 5B) and hematoma volume (*P* = 0.019; Figure 5C).

In Table 3, as compared to patients who did not suffer from END, those at risk of END had significantly elevated percentages of intraventricular hemorrhage (*P* < 0.01) and subarachnoid hemorrhage (*P* < 0.05) and displayed substantially raised Graeb scores (*P* < 0.01), NIHSS scores (*P* < 0.001), hematoma volume (*P* < 0.001), blood glucose levels (*P* < 0.001), and serum Nrf2 levels (*P* < 0.001). Using the binary logistic regression model, which contained the preceding significant variables in univariate analysis, we found that NIHSS score, hematoma volume, and serum Nrf2 levels independently predicted END (Table 4).

**TABLE 3:** Factors associated with early neurologic deterioration (END) after acute intracerebral hemorrhage (ICH).

	Early neurologic deterioration		*P*-value
	Presence	Absence	
Number	30	85	
Age >60 years	21 (70.0%)	47 (55.3%)	0.159
Gender (male/female)	15/15	50/35	0.402
Hypertension	20 (66.7%)	53 (62.4%)	0.673
Diabetes mellitus	7 (23.3%)	11 (12.9%)	0.241
Hyperlipidemia	10 (33.3%)	23 (27.1%)	0.514
Cigarette smoking	12 (40.0%)	27 (31.8%)	0.413
Alcohol drinking	14 (46.7%)	30 (35.3%)	0.271
Pretreatment of statins	9 (30.0%)	16 (18.8%)	0.202
Pretreatment of anticoagulation drugs	1 (3.3%)	5 (5.9%)	1.000
Pretreatment of antiplatelet drugs	6 (20.0%)	10 (11.8%)	0.356
Admission time (h)	11.2 (7.3–15.0)	9.8 (6.2–14.6)	0.586
Blood collection time (h)	12.2 (9.2–16.7)	11.2 (7.5–16.0)	0.649
Systolic arterial pressure (mmHg)	147.4 ± 23.2	151.2 ± 24.0	0.463
Diastolic arterial pressure (mmHg)	85.0 ± 9.3	87.9 ± 11.4	0.207
Hemorrhage locations (lobar/deep)	9/21	17/68	0.260
Intraventricular hemorrhage	17 (56.7%)	21 (24.7%)	0.001
Graeb scores	1 (0–7)	0 (0–0)	< 0.001
Subarachnoid hemorrhage	7 (23.3%)	5 (5.9%)	0.013
NIHSS scores	12 (7–15)	6 (2–8)	< 0.001
Hematoma volume (ml)	28 (15–36)	11 (7–16)	< 0.001
Blood leukocyte count (×10^9^/l)	7.8 ± 2.8	7. 3 ± 2.9	0.364
Blood glucose levels (mmol/l)	12.9 ± 3.7	10.4 ± 3.8	0.003
Serum Nrf2 levels (ng/ml)	15.3 (8.8–22.7)	8.0 (6.4–10.6)	< 0.001

Variables were presented as count (percentage), mean ± standard deviation, or median (upper-lower quartile) as appropriate, and statistical methods included the Chi-square test, Fisher’s exact test, Student’s *t*-test, and the Mann–Whitney test. NIHSS indicates National Institutes of Health Stroke Scale; Nrf2, nuclear factor erythroid 2-related factor 2.

**TABLE 4:** Results of multivariate analysis for prognostic prediction after acute intracerebral hemorrhage (ICH).

	Odds ratio (95% confidence interval)	*P*-value
Early neurologic deterioration		
NIHSS score	1.276 (1.070–1.522)	0.007
Hematoma volume	1.095 (1.016–1.181)	0.018
Serum Nrf2 levels	1.125 (1.027–1.232)	0.011
90-day poor prognosis		
NIHSS score	1.601 (1.239–2.067)	0.001
Hematoma volume	1.094 (1.036–1.156)	0.001
Serum Nrf2 levels	1.217 (1.067–1.387)	0.013

NIHSS indicates National Institutes of Health Stroke Scale; Nrf2, nuclear factor erythroid 2-related factor 2.

### Serum nuclear factor erythroid 2-related factor 2 levels and development of poor 90-day prognosis

**FIGURE 6:**
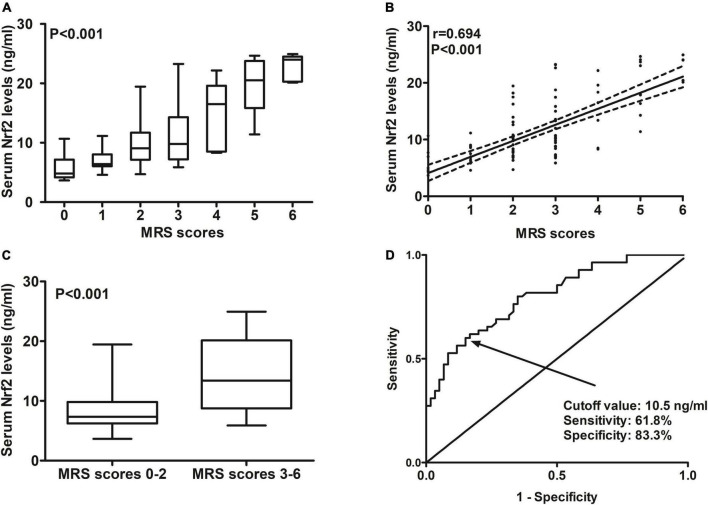
Predictive ability with respect to serum nuclear factor erythroid 2-related factor 2 (Nrf2) levels for post-stroke 90-day poor prognosis after acute supratentorial intracerebral hemorrhage (ICH). **(A)** Comparisons of serum Nrf2 levels among subgroups based on modified Rankin scale (mRS) scores. **(B)** Relationship between serum Nrf2 levels and mRS scores. **(C)** Comparison of serum Nrf2 levels between patients with mRS scores 3–6 and those with scores 0–2. **(D)** Discriminatory capability of serum Nrf2 levels for post-stroke 90-day poor prognosis under the receiver operating characteristic (ROC) curve. Serum Nrf2 levels were intimately correlated with mRS scores and were markedly higher in patients with mRS scores 3–6 than in those with scores 0–2 (all *P* < 0.001). Serum Nrf2 levels statistically significantly predicted 90-day poor prognosis after acute stroke (*P* < 0.001). Nrf2 indicates nuclear factor erythroid 2-related factor 2; mRS, modified Rankin scale.

**FIGURE 7:**
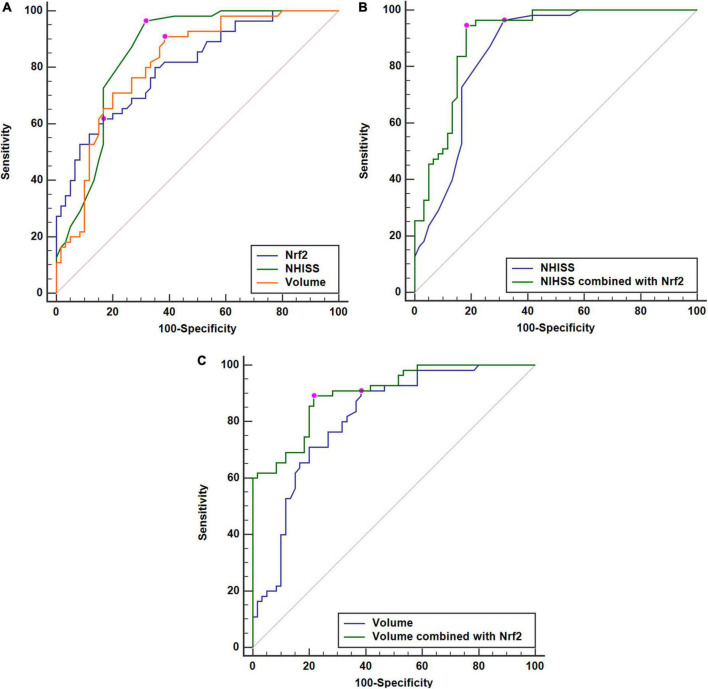
Comparisons of discriminatory capabilities for post-stroke 90-day poor prognosis among serum nuclear factor erythroid 2-related factor 2 (Nrf2) levels, National Institutes of Health Stroke Scale (NIHSS) scores, and hematoma volume after acute supratentorial intracerebral hemorrhage (ICH). **(A)** Comparisons of areas under curve for post-stroke 90-day poor prognosis among serum Nrf2 levels, NIHSS scores, and hematoma volume after acute supratentorial intracerebral hemorrhage (ICH). **(B)** Comparison of areas under curve for post-stroke 90-day poor prognosis between serum Nrf2 levels combined with NIHSS scores and NIHSS scores after acute supratentorial ICH. **(C)** Comparison of areas under curve for post-stroke 90-day poor prognosis between serum Nrf2 levels combined with hematoma volume and hematoma volume after acute supratentorial ICH. Serum Nrf2 levels had similar area under curve, as compared to NIHSS scores and hematoma volume (both *P* > 0.05). Moreover, serum Nrf2 levels profoundly improved areas under curve of NIHSS scores and hematoma volume (both *P* < 0.05). Poor prognosis was defined as modified Rankin Scale (mRS) scores 3–6. Nrf2 indicates nuclear factor erythroid 2-related factor 2; NIHSS, National Institutes of Health Stroke Scale.

At 90 days after ICH, mRS scores ranged from 0 to 6, with a median value of 2 (lower–upper quartiles 1–3). Among them, 10 patients had mRS score 0; 21, mRS score 1; 29, mRS score 2; 33, mRS score 3; 7, mRS score 4; 10, mRS score 5; and 5, mRS score 6. In total, 55 patients experienced a poor 90-day prognosis. In Figure 6A, serum Nrf2 levels were substantially different among subgroups divided by mRS scores, with higher levels in mRS score 6 subgroup and lowest levels in mRS score 0 subgroup (*P* < 0.001). In Figure 6B, serum Nrf2 levels were significantly correlated with mRS scores (*P* < 0.001). In addition, poor prognosis patients had markedly higher serum Nrf2 levels than other remainders (*P* < 0.001; Figure 6C). Under the ROC curve, serum Nrf2 levels >10.5 ng/ml differentiated between poor prognosis patients and good prognosis ones with medium–high sensitivity and specificity values (Figure 6D). In Figure 7A, under the ROC curve, the discriminatory capability of serum Nrf2 levels was similar to those of NIHSS scores and hematoma volume (both *P* > 0.05). Moreover, serum Nrf2 levels substantially enhanced the predictive abilities of NIHSS scores (*P* = 0.034; Figure 7B) and hematoma volume (*P* = 0.024; Figure 7C).

Just as displayed in Table 5, as compared to patients without risk of poor prognosis, those, who were likely to experience poor prognosis, had significantly raised percentages of intraventricular bleedings and subarachnoid bleedings (both *P* < 0.01) and were more prone to exhibit significantly increased NIHSS scores (*P* < 0.001), hematoma volume (*P* < 0.001), blood glucose levels (*P* < 0.05), and serum Nrf2 levels (*P* < 0.001). The above significant variables were forced into the binary logistic regression model, and as a subsequence, NIHSS scores, hematoma volume, and serum Nrf2 levels were retained as the independent predictors of post-stroke 90-day poor prognosis (Table 4).

**TABLE 5:** Factors associated with 90-day functional outcome after acute intracerebral hemorrhage (ICH).

Variables	MRS scores 3–6	MRS scores 0–2	*P*-value
Number	55	60	
Age >60 years	37 (67.3%)	31 (51.7%)	0.089
Gender (male/female)	27/28	38/22	0.124
Hypertension	36 (65.5%)	37 (61.7%)	0.673
Diabetes mellitus	8 (14.5%)	10 (16.7%)	0.754
Hyperlipidemia	15 (27.3%)	18 (30.0%)	0.747
Cigarette smoking	17 (30.9%)	22 (36.7%)	0.515
Alcohol drinking	22 (40.0%)	22 (36.7%)	0.713
Pretreatment of statins	13 (23.6%)	12 (20.0%)	0.637
Pretreatment of anticoagulation drugs	2 (3.6%)	4 (6.7%)	0.681
Pretreatment of antiplatelet drugs	8 (14.5%)	8 (13.3%)	0.851
Admission time (h)	10.5 (7.4–15.2)	9.3 (6.2–14.2)	0.258
Blood collection time (h)	12.2 (9.3–17.0)	10.8 (7.3–15.7)	0.214
Systolic arterial pressure (mmHg)	150.1 ± 24.4	150.3 ± 23.3	0.956
Diastolic arterial pressure (mmHg)	87.0 ± 10.6	87.3 ± 11.2	0.903
Hemorrhage locations (lobar/deep)	15/40	11/49	0.252
Intraventricular hemorrhage	25 (45.5%)	13 (21.7%)	0.007
Graeb scores	0 (0–5)	0 (0–0)	0.086
Subarachnoid hemorrhage	10 (18.2%)	2 (3.3%)	0.009
NIHSS scores	9 (7–12)	4 (2–7)	< 0.001
Hematoma volume (ml)	21 (13–28)	9 (5–13)	< 0.001
Blood leukocyte count (×10^9^/l)	7.9 ± 3.0	7.0 ± 2.7	0.094
Blood glucose levels (mmol/l)	11.8 ± 3.9	10.3 ± 3.8	0.042
Serum Nrf2 levels (ng/ml)	13.4 (8.8–19.9)	7.4 (6.2–9.8)	< 0.001

Variables were presented as count (percentage), mean ± standard deviation, or median (upper-lower quartile) as appropriate, and statistical methods included the Chi-square test, Fisher’s exact test, Student’s *t*-test, and the Mann–Whitney test. NIHSS indicates National Institutes of Health Stroke Scale; Nrf2, nuclear factor erythroid 2-related factor 2; mRS, modified Rankin scale.

## Discussion

To the best of our knowledge, no data are available regarding the relationship between circulating Nrf2 levels and severity in addition to the prognosis of patients with acute brain injury. Our study mainly found such results: (1) Serum Nrf2 levels were profoundly higher in ICH patients than those in healthy controls; (2) there was a dynamic change in serum Nrf2 levels of ICH patients, showing that its levels increased within 6 h, peaked at 24 h, and decreased gradually; (3) serum Nrf2 levels not only were closely correlated with NIHSS scores and hematoma volume using the Spearman test, but also were independently related to NIHSS scores and hematoma volume using multivariable linear regression analysis; (4) serum Nrf2 levels, which were considered as whether the categorical or continuous variable, were highly correlated with 90-day mRS scores after ICH; (5) serum Nrf2 emerged as an independent predictor of END and 90-day poor prognosis; (6) serum Nrf2 levels had similar prognostic predictive ability for END and 90-day poor prognosis after hemorrhagic stroke, as compared to NIHSS scores and hematoma volume; and (7) serum Nrf2 levels substantially improved the predictive efficiency of NIHSS scores and hematoma volume. In a word, serum Nrf2 levels, in close correlation with hemorrhagic severity, independently predicted END and 90-day poor prognosis after ICH, indicating serum Nrf2 may be a promising biochemical marker for facilitating severity assessment and prognosis prediction after acute ICH.

Accumulating evidence has shown that inflammation and oxidative stress participate in the progression of acute brain injury following ICH (Wilkinson et al., 2018). Nrf2, which is identified as a key transcriptional factor, can modulate antioxidant response element-regulated genes (Tonelli et al., 2018). Reportedly, increasing Nrf2 activity could protect against cerebral ischemia *in vivo* (Son et al., 2010). Several animal studies have identified a protective role of Nrf2 in ICH (Wang et al., 2007; Zhao et al., 2007, 2015a,2015b). Specifically, as compared to wild-type mice, Nrf2-knockout mice had significantly increased injury volume, leukocyte infiltration, production of reactive oxygen species (ROS), DNA damage, and cytochrome c release after intracerebral injection of collagenase (Wang et al., 2007). Also, a study of Nrf2-knockout mice has demonstrated that Nrf2 may play an important role in modulating microglia function and hematoma clearance (Zhao et al., 2015a). In a pre-clinical study investigating dimethyl fumarate, a substance for the treatment of multiple sclerosis, as therapy for ICH, Nrf2 was considered as a key factor involved in the protective effects of dimethyl fumarate on ICH (Zhao et al., 2015b). Sulforaphane-activated Nrf2 could obviously reduce neutrophil count, oxidative damage, and behavioral deficits in rats with ICH, and Nrf2-deficient mice had more severe neurologic deficits after ICH (Zhao et al., 2007). Overall, Nrf2 may be a protective factor in acute brain injury after ICH, and recruitment of the antioxidative defense system may be one of its important mechanisms.

Nuclear factor erythroid 2-related factor 2 can be chiefly expressed in neuronal cells. Brain Nrf2 expressions were significantly increased at 2 h and peaked at 8 h of reperfusion in mice after transient middle cerebral artery occlusion (Tanaka et al., 2011). In ICH rat brain, Nrf2 expressions were substantially elevated at 2 h with a peak at 24 h (Shang et al., 2013). Also, there was a significant elevation of expression of Nrf2 in brain tissues after human cerebral cortex contusion (Guo et al., 2019). In the current study, there was a significant enhancement in serum Nrf2 levels after ICH, as compared to healthy controls, and serum Nrf2 levels increased within 6 h, peaked at 24 h, and decreased gradually. Presumably, Nrf2 in the peripheral blood may be at least partially derived from injured brain tissues after ICH. In consideration of its cytoprotective effects, Nrf2 released from brain tissues may be a compensatory response to brain inflammatory and oxidative damage after ICH. Hence, Nrf2 may play an important role in acute brain injury after ICH.

Experimentally, Nrf2 expressions in brain tissues had close correlation with brain edema and neurologic deficit after ICH (Shang et al., 2013). In our study, serum Nrf2 levels, in independent correlation with hemorrhagic severity indicated by NIHSS scores and hematoma volume, were independently predictive of END and poor neurologic function prognosis after ICH. Interestingly, serum Nrf2 levels were of significant efficiency in predicting END and poor prognosis after ICH under the ROC curve. Of note, the predictive ability of its combination with NIHSS scores or hematoma volume substantially exceeded that of NIHSS scores or hematoma volume alone. Taken together, our data are supportive of the hypothesis that serum Nrf2, as a potential prognostic biomarker, may be of clinical value in the treatment of ICH.

In the current study, it was confirmed that increased serum Nrf2 levels were independently associated with hemorrhagic severity and poor clinical outcome after acute ICH. However, the average turnaround time for laboratory results in the emergency departments is between 30 and 40 min currently. Nevertheless, it holds great potential, as it were, to become more time efficient and, more importantly, to have another way to predict outcome will not only help the clinicians but also the family to make decisions as well as necessary adjustments to care for the recovering patient.

There are several limitations in the current study. First, the sample size is small in this study, so this is a pilot study and makes a preliminary conclusion that serum Nrf2 levels may be associated with severity and prognosis after acute ICH. A larger cohort study is warranted to validate the conclusions. Second, we do not do routine work to complete two times head computed tomography scans within 24 h after ICH. Subsequently, there is a paucity of data available regarding early hematoma growth. So, the results may be biased and the conclusions should be explained cautiously. Third, we performed a clinical epidemiological investigation regarding the relationship between serum Nrf2 levels and severity plus poor prognosis after acute ICH. Nevertheless, a further study using some bioinformatics analyses, computational biological analyses, PCR, and western blotting assay may provide some information to identify which oxidative stress-related signaling pathways Nrf2 may regulate in the pathogenesis of acute ICH. Last, our study did not use the ROS detection assay for determining ROS, so there is not enough evidence supportive of the notion that Nrf2 may regulate ROS in ICH. Hence, the role of Nrf2 in pathophysiological processes of ICH remains to be confirmed.

## Conclusion

To the best of our knowledge, our study, for the first time, measured circulating Nrf2 levels of humans with acute brain injury and further investigated the relation of serum Nrf2 levels to illness severity and prognosis after ICH. Independent correlation is ascertained between serum Nrf2 levels and NIHSS scores in addition to hematoma volume, and also, independent association is discerned between serum Nrf2 levels and END plus 90-day poor prognosis in this cohort of ICH patients. Hypothetically, Nrf2 may participate in pathophysiological processes of acute brain injury after ICH, and serum Nrf2 may be a promising biomarker, which could be of clinical significance in severity assessment and prognosis prediction of ICH.

## Data availability statement

The raw data supporting the conclusions of this article will be made available by the authors, without undue reservation.

## Ethics statement

The studies involving human participants were reviewed and approved by The Quzhou Affiliated Hospital of Wenzhou Medical University. The patients/participants provided their written informed consent to participate in this study.

## Author contributions

All authors listed have made a substantial, direct, and intellectual contribution to the work, and approved it for publication.

## Abbreviations

END: early neurologic deterioration
CT: computerized tomography
ICH: intracerebral hemorrhage
NIHSS: National Institutes of Health Stroke Scale
ROC: receiver operating characteristic
mRS: modified Rankin scale
Nrf2: nuclear factor erythroid 2-related factor 2
SD: standard deviation
ROS: reactive oxygen species.
